# The Mechanism of Onychomadesis (Nail Shedding) and Beau’s Lines Following Hand-Foot-Mouth Disease

**DOI:** 10.3390/v11060522

**Published:** 2019-06-06

**Authors:** Hsiu-Hui Chiu, Ming-Tsan Liu, Wen-Hung Chung, Yu-Shien Ko, Chin-Fang Lu, Cheng-Che E. Lan, Chun-Wei Lu, Kai-Che Wei

**Affiliations:** 1 Department of Dermatology, Pingtung Christian Hospital, Pingtung 90059, Taiwan; chioush88@gmail.com; 2 Department of Dermatology, E-Da Cancer Hospital, Kaohsiung 82445, Taiwan; 3 College of Medicine, I-Shou University, Kaohsiung 82445, Taiwan; 4 Center for Research, Diagnostics and Vaccine Development, Centers for Disease Control, Taipei 100, Taiwan; mtliu@cdc.gov.tw; 5 Department of Dermatology, Drug Hypersensitivity Clinical and Research Center, Chang Gung Memorial Hospital, Taoyuan 333, Taiwan; chung1@cgmh.org.tw; 6 College of Medicine, Chang Gung University, Taoyuan 333, Taiwan; 7 Cardiovascular Division, Microscope Core Laboratory, Chang Gung Memorial Hospital, Taoyuan 333, Taiwan; c12037@cgmh.org.tw; 8 Department of Dermatology, Research Center of Cutaneous Disorders, Chung Shan Hospital, Taipei 106, Taiwan; dermlu.yyf@gmail.com; 9 Department of Dermatology, Kaohsiung Medical University Hospital, Kaohsiung 80708, Taiwan; laneric@gmail.com; 10 Department of Dermatology, Faculty of Medicine, College of Medicine, Kaohsiung Medical University, Kaohsiung 80708, Taiwan; 11 Department of Dermatology, Kaohsiung Veterans General Hospital, Kaohsiung 81362, Taiwan

**Keywords:** onychomadesis, Coxsackievirus A6, hand-foot-mouth disease

## Abstract

Background: Nail changes, including onychomadesis (nail shedding) and Beau’s line, following hand-foot-mouth disease (HFMD) are a common emergence at the stage of late complications of HFMD. However, the exact mechanism is still unknown. Therefore, we conducted this study to elucidate the mechanism of nail changes following HFMD. Methods: We collected 11 patients suffering from onychomadesis following HFMD. Nail samples from all of them were collected. Real time reverse transcription polymerase chain reaction (RT-PCR) and sequencing for human enteroviruses (HEV) were performed. Throat swabs for RT-PCR and sequencing for HEV were performed for three cases. Results: RT-PCR demonstrated the presence of Coxackievirus A6 (CVA6) in nail samples from three patients and one with Echovirus. Conclusion: In conclusion, we believe that the major cause of onychomadesis following HFMD is that certain novel viruses, mostly CVA6, are virulent and may damage nail matrix. Direct injury caused by cutaneous lesions of HFMD around nail matrix is a minor cause. There are still other virulent HEV which may result in onychomadesis. In addition, the novel strain of CVA6 also causes atypical clinical presentations, such as adult involvement and delayed-onset palmar and plantar desquamation. Physicians should be familiar with atypical presentations caused by novel viruses to avoid misdiagnosis and even inform patients of the possibility of onychomadesis that may take place weeks later to reassure patients.

## 1. Introduction

Onychomadesis (nail shedding) is defined as the proximal nail plate detached from the proximal nail fold by a whole thickness sulcus. The causes include mechanical trauma, autoimmune diseases, major medical illness, medications, idiopathic and infections [1,2,3,4]. Among infections, hand–foot–mouth disease (HFMD) is most common [3,4]. Growth arrest of nail matrix following HFMD was first reported by Clementz et al. in 2000, in Chicago [1]. Subsequently, in 2001, Bernier et al. reported the first instance in Europe [2]. Strangely enough, related reports were not published until 2009, after which around 50 have appeared. Today, onychomadesis has been listed as one of the late cutaneous features of HFMD [3]. However, the mechanism of onychomadesis following HFMD is still unknown [4]. There are several hypotheses explaining this temporary inhibition of nail matrix proliferation. The first is severe systemic impact and the second, direct injuries of the nail matrix by cutaneous lesions of HFMD, such as vesicles, around the nail matrix; finally, there are specific novel variants of human enteroviruses (HEV) which are virulent, with new kinds of virus–host interactions leading to nail matrix dysfunction. A detailed discussion appears below. 

## 2. Materials and Methods

From 2010 to 2016, we gathered 11 onychomadesis cases from Chang Gung Memorial Hospital, and Chung Shan Hospital in Taiwan (approval code: IRB code: 103-7459A3). Among them, three came to the hospital at the acute stage of viral infection (Cases 9, 10, and 11). One was our dermatologist (Case10, our correspondence author) who had gotten HFMD in our emergency department and one was his daughter (Case 9). The other 8 patients visited our clinic for the presence of onychomadesis as a consequence of HFMD. The patients’ characteristics are summarized in Table 1. We collected clinical specimens including throat swabs from patients within acute stage of HFMD and nail sloughing resulting from onychomadesis. 

### 2.1. Clinical Sample Preparation

Throat swabs of the patients were collected while they visited our clinic and preserved in a viral transport medium of sterilized tissue culture tube (Coring^®^) and sent out for RT-PCR.

The shedding nail plates of onychomadesis had been first removed from the affected finger or toe and then separated into 3 groups. The first group was treated with the routine process as the above-mentioned throat swabs. The other 2 groups of specimens were either treated with 20% KOH solution for one hour for the digestion of keratinocytes and keratins. After the treatment, samples were all preserved in the viral transport medium for RT-PCR. 

### 2.2. Viral Culture and Virus DNA Detection in Clinical Samples

The total virus nucleic acids from clinical samples were extracted by using QIAamp Viral RNA Mini Kits, according to the manufacturer’s instructions (Qiagen, Santa Clara, CA, USA) and the MagNa Pure LC extraction system (Roche). For HEV detection, extracted RNA was first analyzed to determine the presence of HEV by one-step real-time RT-PCR [5]. The type of HEV was determined by conventional RT-PCR using QIAGEN OneStep RT-PCR Kit (Qiagen) with primers targeting 5’UTR (forward primer: 5′-CAAGCACTTCTGTNWCCCGG-3′; reverse primer: 5′-GAAACACTGGACACCCAAAGTAGT-3′) or targeting VP1 gene as described previously [6]. The PCR products were confirmed by agarose electrophoresis and were then sequenced by using a BigDye Terminator Ready Reaction Cycle Sequencing Kit and an automated sequencer ABI 3730 (Applied Biosystems, Foster City, CA, USA). Obtained sequences were analyzed by using BLAST in GenBank and confirmed by phylogenetic analysis.

### 2.3. Phylogenetic Analysis 

Multiple sequence alignments and phylogenetic analysis were performed on the basis of nucleotide sequences using the software MEGA6 [7] and BioEdit (http://www.mbio.ncsu.edu/BioEdit/bioedit.html). A phylogenetic tree was constructed by the neighbor-joining method and 1000 bootstrap replications were performed to evaluate the reliabilities.

We analyzed the shedding plantar desquamation from his right heel about 38 days after acute infection (Figure 2b) and the nail plate neighboring nail matrix of onychomadesis on his right thumbnail about 69 days after acute infection (Figure 2c).

## 3. Result

The intervals from acute viral infection episode to notification of nail change are from one month to 96 days (mean: 53.4 days). Five of the patients (45%) were adults (>18 years). 

**Figure 1:**
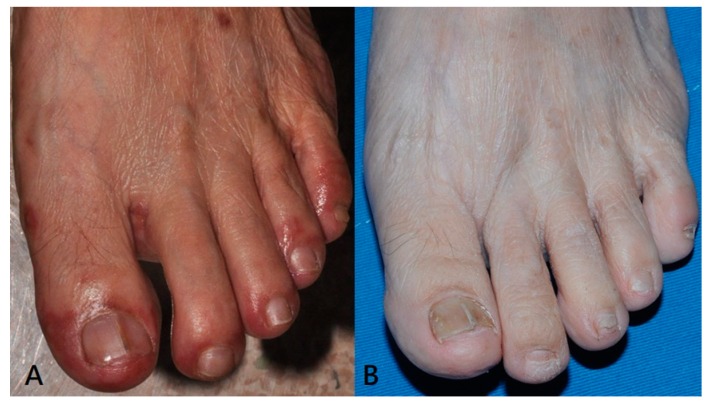
(**A**) In the acute stage, the cutaneous lesions, such as erythematous papules and pustules, around the nail matrix of the first, fourth and fifth toenails of left foot are relatively prominent than on the second and third toe; (**B**) about two months later, onychomadesis on the second and third toe of left foot.

In Case 11, about two months after acute infection, onychomadesis on the left second and third toenails was noted. Comparing with the photo of acute infection stage, cutaneous lesions around the nail matrix on the first, fourth and fifth toes are more prominent. Cutaneous lesions around nail matrix on second and third toe are relatively mild (Figure 1). It seems that the appearance of onychomadesis is not exactly associated with the cutaneous lesions around nail matrix in the acute infection stage. 

**Figure 2:**
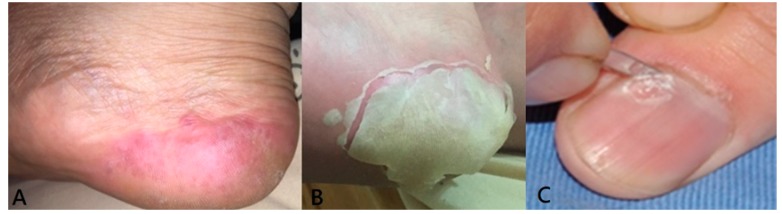
(**A**) In the acute stage, multiple erythematous vesicles on right heel; (**B**) plantar desquamation on right heel, about 38 days later; (**C**) sampling nail plate neighboring to nail matrix of onychomadesis nail from patient’s right thumbnail, about 69 days later.

In Case 10, delay onset plantar desquamation was noted on the right heel about 38 days after acute infection stage (Figure 2b). This is an unusual finding in classical HFMD.

**Figure 3:**
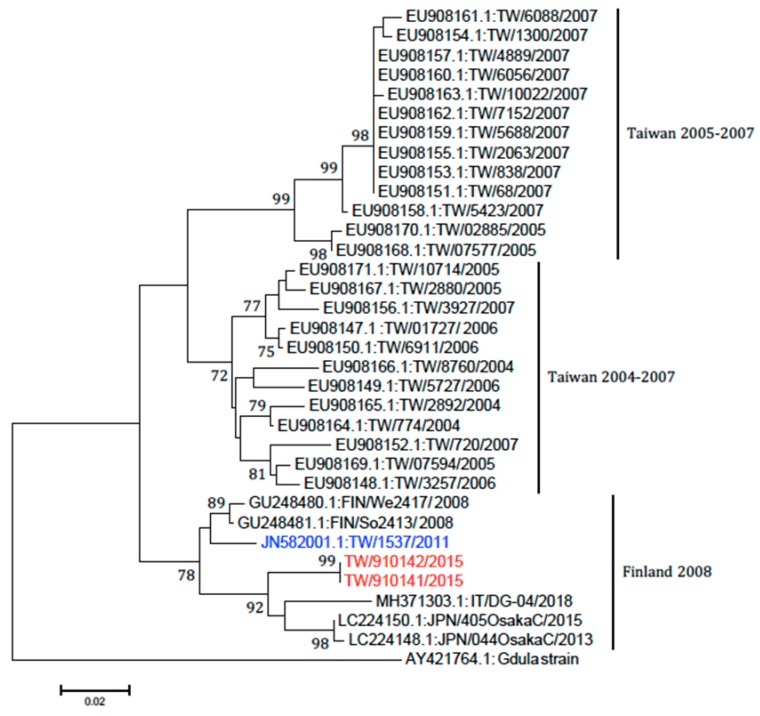
Phylogenetic analysis of Coxsackievirus A6 strains. The partial VP1 gene sequences (377 nucleotides) of our A6 strains (TW/910141 and TW/910142 in red) and the strains in Taiwan from 2004 to 2011 along with six sequences obtained from Genbank (two from Finland, two from Japan, one from Italy, and the prototype strain, Gdula) were used for the phylogenetic analysis. One strain (highlighted with blue) is identical to Finland strain we published in 2013.

RT-PCR demonstrated the presence of Coxsackievirus A6 (CVA6) in nail plates from three patients and one with Echovirus, where the specimens were treated with 20% KOH and viral transport medium. However, viral RNA sequence could not be detected in the specimens treated with acetic acid. The CVA6 is a novel variant, previously identified as the causative pathogen for patients with severe cutaneous adverse reaction-like presentation in Taiwan, in 2013 [8]. This novel variant of CVA6 shares a higher similarity to that of CVA6 strains found in Finland in 2008 than that of Taiwan isolates (Taiwan 2004–2007) [8] (Figure 3). The viral sequence was attached in the Supplementary File S1. 

## 4. Discussion

Onychomadesis and Beau’s line (transverse groove on nail plate) are both presentations of nail matrix arrest and they share the same causes. Onychomadesis is usually a more severe form than Beau’s line. Nail matrix arrest following HFMD is currently a well-recognized phenomenon. The latency period for onychomadesis following HFMD was one to two months (mean: 40 days) [9]. However, the mechanism involved remains unclear today. Several hypotheses explained this temporary inhibition of nail matrix proliferation. First, HFMD imposes severe systemic impact. However, not all nails are affected. On average, each patient sheds only four nails [9]. In addition, there is no relationship linking onychomadesis with the severity of HFMD [9]. No serious complication of HFMD was mentioned in previous reports [9]. These findings do not support the hypothesis that severe systemic impact causes nail matrix arrest. Second, direct injuries of the nail matrix from the cutaneous lesions of HFMD, such as vesicles or pustules, around the nail matrix may be responsible. Almost all previous reports describing onychomadesis were retrospective. Shikuma et al. prospectively observed a case of HFMD in which onychomadesis developed only in the fingernails and toenails following the healing of cutaneous lesions around the affected nails [10]. Nail matrix arrest due to direct injuries from skin lesions around nail matrix was postulated as one of the causes of onychomadesis following HFMD. However, it is hard to explain as HFMD is an ancient disease but onychomadesis is a relative new complication. We do believe that direct injuries caused by the cutaneous lesions of HFMD around nail matrix is one of the causes of onychomadesis, although it appears to be not the only one. In our study, we prospectively observed a patient (Case 11). Onychomadesis developed in the second and third toenails of left foot approximately in the two months after HFMD (Figure 1). In the acute stage, the cutaneous lesions, such as erythematous papules, vesicles and pustules, around the nail matrix of the first, fourth and fifth toenails of the left foot were relatively prominent compared to the second and third toenails. It seems that onychomadesis is not exactly associated with the development of the cutaneous lesions around the nail matrix during the acute stage. Nails of relatively uninvolved nail matrix are still possible candidates for developing onychomadesis. Our observation further reinforce that other causes are involved in addition to the direct injuries caused by cutaneous lesions of HFMD.

After reviewing the literature, we noted that certain viral strains are associated with onychomadesis following HFMD, some even leading to outbreak, but others are not. This obviously cannot be explained simply by direct injuries caused by cutaneous lesions of HFMD. So, the third hypothesis is that specific novel variants of HEV are virulent and new kinds of virus–host interactions may lead to nail matrix dysfunction. It has been postulated that CVA6 may be the major subtype associated with onychomadesis following HFMD [4]. In our study, RT-PCR demonstrated the presence of CVA6 in three cases, and this CVA6 is identical to the one previously identified in 2013, and they share a high homology with the one found in Finland in 2008 [8]. During the fall of 2008, an outbreak of HFMD with onychomadesis occurred in Finland and CVA6 was identified from shed nails [11]. These facts support the hypothesis that a virulent new virus may damage the nail matrix and cause onychomadesis. In our study, Echovirus was identified in one case. This means that other kinds of HEVs may be capable of causing onychomadesis. Our results are comparable with a previous study in Taiwan in 2010. The incidence of onychomadesis after CVA6 infection was 37% when compared to 5% in the case of non-CVA6 causative strains [12]. 

In our study, viruses were detected via PCR, even 50–69 days after the acute stage of HFMD, although this was not entirely unexpected. As we know, nails can record months prior to specimen collection. It is well known that nails can be useful specimens for detecting an exposure to toxic elements, such as heavy metals, that occurred in the month or more prior to the specimen collection. The nail plate is a fully keratinized structure. It results from maturation and differentiation of the epithelial nail matrix cells. If the epithelial nail matrix cells were infected by a virus, the viral particles will exist in the nail plate. Since viruses are obligate intracellular parasites that can be maintained only inside of living cells, nail plates are composed only of specialized keratin. We believe that it does not represent persistent infection; it merely indicates that the host has been infected by this virus in the past. In our study, virus cultures were performed in two cases (Cases 9 and 10). There was no cytopathic effect in either of them, which further reinforces our hypothesis. These viral particles in nail plates are non-living and not infectious. 

This novel strain of CVA6 has also been reported previously in atypical clinical presentations, widespread mucocutaneous bullous reactions, and even mimics severe cutaneous adverse reactions [8]. We believe that the evolution of novel CVA6 leads to changes of the virus’s characteristics. This leads to new virus-host interactions and results in different clinical presentations in the host, not only in the atypical presentation during the acute infection stage, but also in the post-syndrome nail changes. This can reasonably explain why HFMD is an ancient disease but onychomadesis following HFMD has only been noted in the past 20 years or so. 

Our study had some limitations. Biopsy from the nail matrix may possibly cause permanent injury. Since onychomadesis following HFMD may recover spontaneously without any sequela, it is not ethical to perform nail matrix biopsies on patients. Therefore, we cannot observe cells of the nail matrix directly via TEM. 

To sum up, direct injuries by cutaneous lesions of HFMD around the nail matrix is a possible cause for virus-associated onychomadesis. Since almost all studies are retrospective, we do not know the proportion of cutaneous lesions around the nail matrix preceding onychomadesis. However, it seems to be playing a limited role because onychomadesis following HFMD has never been recorded before 2000 in the literature. Certain novel viruses, including CVA6, are virulent and may damage the nail matrix and lead to the development of onychomychosis. This seems to be the major cause.

On the other hand, is this new kind of virus-host interaction associated with the susceptibility of the host, similar to drug eruptions? Patients take the same drug but only certain susceptible patients may lead to drug eruptions. In a Taiwanese report, the incidence of onychomadesis after CVA6 infection was 37% [12]. It seems that the view of susceptibility of host possibly exists; further studies are needed to clarify this hypothesis in the future. 

Although Coxsackievirus A16 has traditionally been the primary strain causing HFMD, CVA6 has become a major cause of HFMD outbreak worldwide in recent years [12]. The first reported CVA6 associated HFMD outbreak was in Finland in 2008 [11,13,14]. Subsequently, similar strains were detected in Singapore, Taiwan, Japan, Spain, France, China, India, United States, Thailand, Cuba, New Zealand, UK and Denmark [13,14]. HFMD caused by CVA6 often presents atypically. Classical HFMD mainly affects pediatric papulation and is characterized by lesions of the oral mucosa, palms, and soles, lasting for 7 to 10 days. However, CVA6 is more virulent. It occurs with broader demographics and also affects adult patients [13]. In our study, 45% (5/11) of patients were adults. This percentage is obviously higher than in traditional classical HFMD. In addition, HFMD caused by CVA6 leads to more severe afflictions. Patients often suffer from higher fever, longer duration (typically two weeks), and more severe skin manifestations, including wider distribution of not only vesicles but also large bullae, erosions and ulcers. The condition is easy to misdiagnose. Besides, delayed-onset palmar and plantar desquamation and onychomadesis are unique post-syndrome findings [13,14]. In our study, we did note plantar desquamation on the right heel of Case 10, an unusual finding in classical HFMD (Figure 2b).

In conclusion, we believe that the major cause of onychomadesis following HFMD is that certain novel viruses, mostly CVA6, are virulent and may damage nail matrix. Direct injury caused by cutaneous lesions of HFMD around the nail matrix is a minor cause. 

There are still other virulent HEVs which may result in onychomadesis. In addition, the novel strain of CVA6 also causes atypical clinical presentations, such as adult involvement and delayed-onset palmar and plantar desquamation. Physicians should be familiar with atypical presentations caused by novel viruses to avoid misdiagnosis and even inform patients of the possibility of onychomadesis that may take place weeks later to reassure patients.

## Figures and Tables

**Table 1:** Patients with onychomadesis following HFMD.

Case No.	Age/Sex	Interval after HFMD (days)	Nail Samples for PCR and Sequencing for HEV	Nail Samples for Virus Culture	Throat Swab for PCR and Sequencing for HEV	Throat Swab Sampling Date after HFMD Onset	Nail Samples for Transmission Electron Microscope
1	3/F	30	(−)	ND	ND	ND	ND
2	3/M	50	CVA6	ND	ND	ND	ND
3	4/M	30	(−)	ND	ND	ND	ND
4	5/F	30	(−)	ND	ND	ND	ND
5	15/M	50	(−)	ND	ND	ND	ND
6	45/M	60	(−)	ND	ND	ND	ND
7	30/F	96	(−)	ND	ND	ND	ND
8	69/F	60	(−)	ND	ND	ND	ND
9	1/F	62	CVA6	(−)	CVA6	2nd day	ND
10	31/M	69	CVA6	(−)	CVA6	2nd day	viral particles (+)
11	60/F	50	Echovirus	ND	Echovirus	5th day	ND

Abbreviations: (−) Negative; F: Female; M: Male; CoxsackievirusA6 (CVA6); human enteroviruses (HEV); hand-foot-mouth disease (HFMD); polymerase chain reaction (PCR); ND: Not determined.

## Supplementary Materials

The following are available online at https://www.mdpi.com/1999-4915/11/6/522/s1, File S1: Partial sequence of VP1 and 5’UTR for some isolates.

## References

[1] Clementz G.C., Mancini A.J. (2000). Nail matrix arrest following hand-foot-mouth disease: A report of five children. Pediatr. Dermatol..

[2] Bernier V., Labrèze C., Bury F., Taïeb A. (2001). Nail matrix arrest in the course of hand, foot and mouth disease. Eur. J. Pediatr..

[3] Chiu H.H., Wu C.S., Lan C.E. (2017). Onychomadesis: A Late Complication of Hand, Foot, and Mouth Disease. J. Emerg. Med..

[4] Hardin J., Haber R.M. (2015). Onychomadesis: Literature review. Br. J. Dermatol..

[5] Tamura K., Stecher G., Peterson D., Filipski A., Kumar S. (2013). MEGA6: Molecular Evolutionary Genetics Analysis version 6.0. Mol. Biol. Evol..

[6] Bonzel L., Tenenbaum T., Schroten H., Schildgen O., Schweitzer-Krantz S., Adams O. (2008). Frequent detection of viral coinfection in children hospitalized with acute respiratory tract infection using a real-time polymerase chain reaction. Pediatr. Infect. Dis. J..

[7] Oberste M.S., Maher K., Flemister M.R., Marchetti G., Kilpatrick D.R., Pallansch M.A. (2000). Comparison of Classic and Molecular Approaches for the Identification of Untypeable Enteroviruses. J. Clin. Microbiol..

[8] Chung W.H., Shih S.R., Chang C.F., Lin T.Y., Huang Y.C., Chang S.C., Liu M.T., Ko Y.S., Deng M.C., Liau Y.L. (2013). Clinicopathologic analysis of coxsackievirus a6 new variant induced widespread mucocutaneousbullous reactions mimicking severe cutaneous adverse reactions. J. Infect. Dis..

[9] Chiu H.H., Lan C.C., Wu C.S., Kuo K.C., Chen G.S., Wei K.C. (2016). Onychomadesis following hand-foot-and-mouth disease. Cutis.

[10] Shikuma E., Endo Y., Fujisawa A., Tanioka M., Miyachi Y. (2011). Onychomadesis developed only on the nails having cutaneous lesions of severe hand–foot–mouth disease. Case Rep. Dermatol. Med..

[11] Osterback R., Vuorinen T., Linna M., Susi P., Hyypiä T., Waris M. (2009). Coxsackievirus A6 and hand, foot, and mouth disease, Finland. Emerg. Infect. Dis..

[12] Wei S.H., Huang Y.P., Liu M.C., Tsou T.P., Lin H.C., Lin T.L., Tsai C.Y., Chao Y.N., Chang L.Y., Hsu C.M. (2011). An outbreak of coxsackievirus A6 hand, foot, and mouth disease associated with onychomadesis in Taiwan, 2010. BMC Infect. Dis..

[13] Kimmis B.D., Downing C., Tyring S. (2018). Hand-foot-and-mouth disease caused by coxsackievirus A6 on the rise. Cutis.

[14] Horsten H.H., Kemp M., Fischer T.K., Lindahl K.H., Bygum A. (2018). Atypical Hand, Foot, and Mouth Disease Caused by Coxsackievirus A6 in Denmark: A Diagnostic Mimicker. Acta Derm. Venereol..

