# Supplementary material for: The Mechanism of Onychomadesis (Nail Shedding) and Beau’s Lines Following Hand-Foot-Mouth Disease
Source: Viruses. 2019 Jun 6;11(6):522. doi: 10.3390/v11060522 (PMC6630444; doi:10.3390/v11060522)
Supplement: Supplementary file 1 [file viruses-11-00522-s001.pdf]

Title: Partial sequence of VP1 and 5'UTR for some isolates:

>TW/910142/2015 [organism=Enterovirus] [isolate=coxsackievirus A6] Human coxsackievirus A6 strain VP1 gene, partial CDS

TACCAATGGCAGACCGCTACCAACCCGTCAATATTCGCAAAATTAAGCGATCCACCCCCTCAG
GTGTCTGTCCCGTTTATGTCACCAGCAACAGCCTATCAGTGGTTTTATGATGGTTACCCTACATT
TGGTGAGCACAAACAAGCCACTAATTTACAGTATGGACAGTGCCCTAACAACATGATGGGCCA
TTTTGCCATCCGAACAGTCAGTGAATCTACCACCGGGAAAAATGTCCACGTTCGGGTGTACAT
GAGAATTAAGCACGTGAGAGCTTGGGTACCTAGACCCCTTCGGTCCCAAGCGTATATGGTCAA
GAATTACCCGACATATAGCCAAACAATAACTAACACTGCAACCGATCGTGCAAGCATAAC

>TW/910141/2015 [organism=Enterovirus] [isolate=coxsackievirus A6] Human coxsackievirus A6 strain VP1 gene, partial CDS

TACCAATGGCAGACCGCTACCAACCCGTCAATATTCGCAAAATTAAGCGATCCACCCCCTCAG
GTGTCTGTCCCGTTTATGTCACCAGCAACAGCCTATCAGTGGTTTTATGATGGTTACCCTACATT
TGGTGAGCACAAACAAGCCACTAATTTACAGTATGGACAGTGCCCTAACAACATGATGGGCCA
TTTTGCCATCCGAACAGTCAGTGAATCTACCACCGGGAAAAATGTCCACGTTCGGGTGTACAT
GAGAATTAAGCACGTGAGAGCTTGGGTACCTAGACCCCTTCGGTCCCAAGCGTATATGGTCAA
GAATTACCCGACATATAGCCAAACAATAACTAACACTGCAACCGATCGTGCAAGCATAAC

>TW/913268/2016 [organism=Enterovirus] [isolate=echovirus 30] Human Echovirus E30 strain 5' UTR

ACTTCGAGAAACTTAGTACCACCATGAAAGTTGCGCAGCGTTTCGCTCCGCACGACCCCAGT
GTAGATCAGGCTGATGAGTCACCgCGTTCCTCACGGGTGACCGTGGCGGTGGCTGCGTTGGC
GGCCTGCCCTTGGGGCAACCCAAGGGACGCTTCAATACTGACATGGTGCGAAGAGTCTATTG
AGCTAATTGGTAGTCCTCCGGCCCCTGAATGCGGCTAATCCCAACTGCGGAGCAGATACCCAC
ATACCAGTGGGCAGTCTGTCGTAATGGGCAACTCTGCAGCGGAACCGACTACTTTGGGTGTC
CGTGTTTC
